# Oncocytic Cell Carcinoma of the Thyroid: A Case Report and an Overview of the Diagnosis, Treatment Modalities, and Prognosis

**DOI:** 10.7759/cureus.30298

**Published:** 2022-10-14

**Authors:** Ameena Syed, Sai Aishwarya Vanka, Ivan Escudero, Rana Ismail, Hicham Krayem

**Affiliations:** 1 Department of Internal Medicine, Detroit Medical Center-DMC/Wayne State University/Sinai Grace Hospital, Detroit, USA; 2 Department of Internal Medicine, Albert Einstein College of Medicine-Montefiore Medical Center, Wakefield Campus, New York, USA; 3 Department of Family Medicine, Detroit Medical Center-DMC/Michigan State University College of Medicine, Detroit, USA; 4 College of Osteopathic Medicine, Michigan State University, Detroit, USA

**Keywords:** oncocytic thyroid carcinoma, total thyroidectomy, hürthle cell carcinoma, histopathological examination, prevalence, tracheostomy placement

## Abstract

Cancers of the thyroid gland are uncommon, accounting for 1% of malignant tumors. Oncocytic carcinoma of the thyroid (OCA), previously known as “Hürthle cell” carcinomas, make up 3% to 5% of all thyroid cancers and are extremely rare. In the United States, the incidence of thyroid cancer is approximately 12 per 100,000 per year and increases with age. The prevalence of thyroid cancers in women is estimated to be twice that in men, with the male gender correlating with a worse prognosis.

A definitive diagnosis of OCA is confirmed after complete excision and histopathological examination. OCA is an aggressive tumor with an exceedingly low incidence, a high risk of metastasis, and a poor prognosis. The mainstay of therapy for OCA is surgery. In order to recognize and treat the disease as early as possible, healthcare providers must consider the probability of OCA in patients presenting with a thyroid mass.

We hereby present a case of OCA identified incidentally status post tracheostomy and subsequent biopsy. We have an opportunity to review this disease with the hope of improving outcomes by raising awareness and with early recognition.

## Introduction

Thyroid gland cancers are sporadic, accounting for about 0.5% to 1% of all malignant tumors [1]. OCA, sometimes called oxyphilic carcinoma, is a rare condition accounting for 3% to 5% of all thyroid cancers [1,2-5]. In the United States, the incidence of thyroid cancer is about 12 per 100,000 per year, and the incidence increases with age. Oncocytes (also known as Ashkenazy cells and oxyphil cells) are large polygonal cells found in the thyroid and parathyroid glands. They have an abundance of eosinophilic cytoplasm secondary to the increased mitochondrial content.

OCAs, formerly known as “Hürthle cell” carcinomas, represent a category of thyroid tumors with genetically and clinically distinguished features that differ from non-oncocytic thyroid cancers [6]. The term “Hürthle cell” is a misnomer; hence, the term is discouraged. According to the World Health Organization classification for thyroid cancers 2022 (5th edition), oncocytic carcinoma is a distinct entity with explicit recognition that indicates oncocytic follicular cell-derived neoplasms (composed of > 75% oncocytic cells). It lacks the characteristic nuclear features of papillary thyroid cancers (PTC) and high-grade features (necrosis and ≥ 5 mitoses per 2 mm^2^). High-grade follicular-cell-derived cancers comprise both high-grade differentiated thyroid carcinomas and poorly differentiated carcinomas [6].

The median age at diagnosis of OCA is approximately 55 years, which is 10 years more than that of follicular malignancies (45 years). The prevalence of thyroid cancer in women is estimated to be twice that of men, with males having a poorer prognosis than females. The prognosis is worse for those over 65 years of age [7]. Here, we present a case of OCA identified inadvertently in an older woman.

## Case presentation

**Figure 1:**
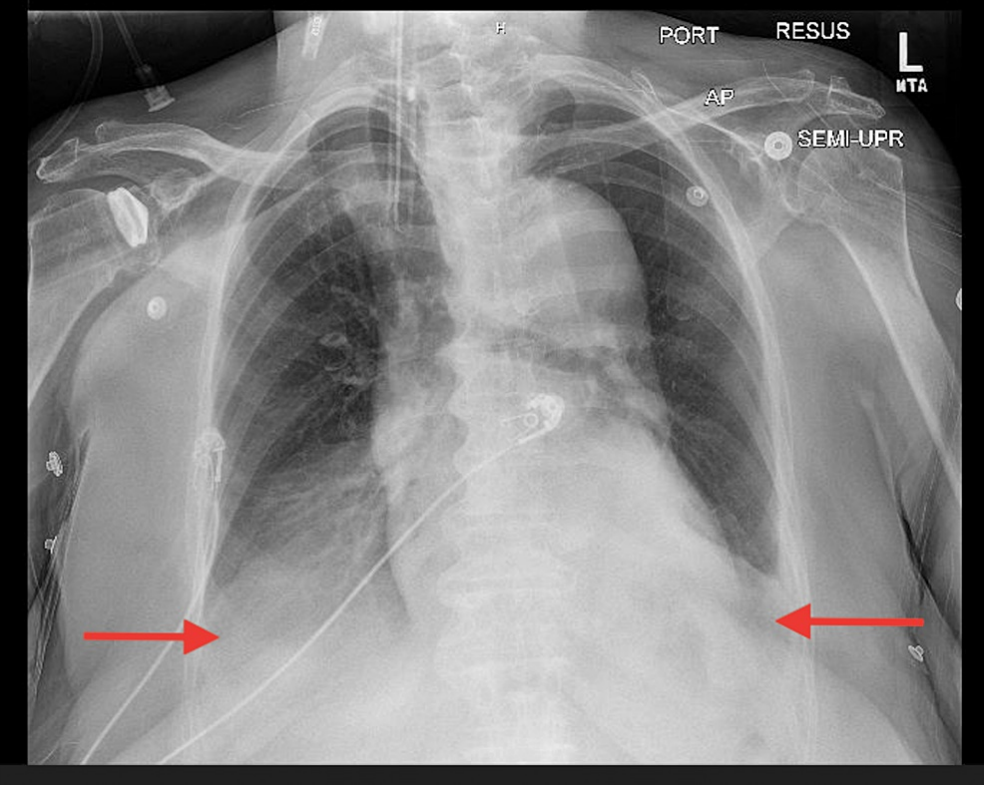
Chest X-ray revealing small bilateral effusions

**Figure 2:**
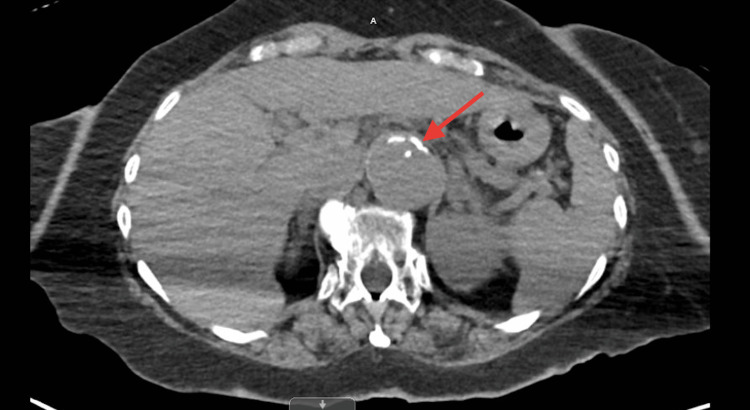
A CT angiogram of the abdomen and thorax revealing a stable type B aortic dissection

**Figure 3:**
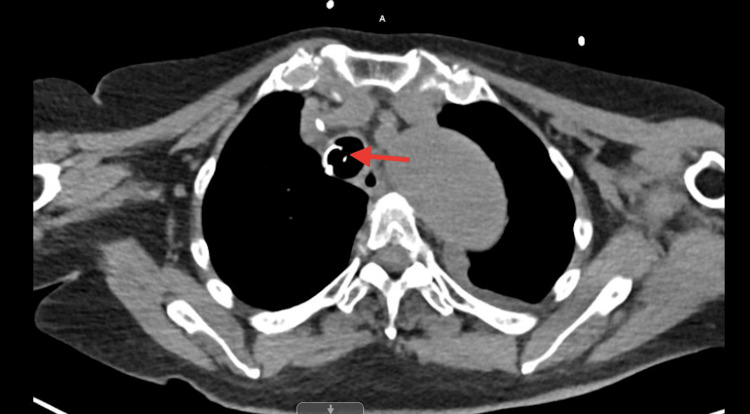
A CT angiogram of the abdomen and thorax revealing a stable type B aortic dissection

**Figure 4:**
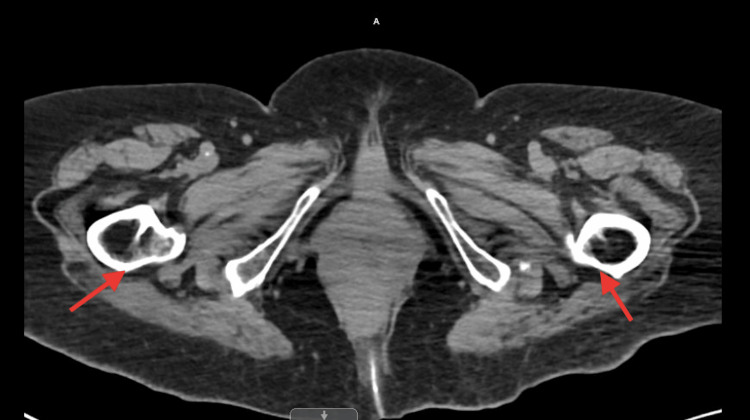
A CT angiogram of the abdomen showing heterogeneous masses in both proximal femurs, consistent with bony metastases

**Figure 5:**
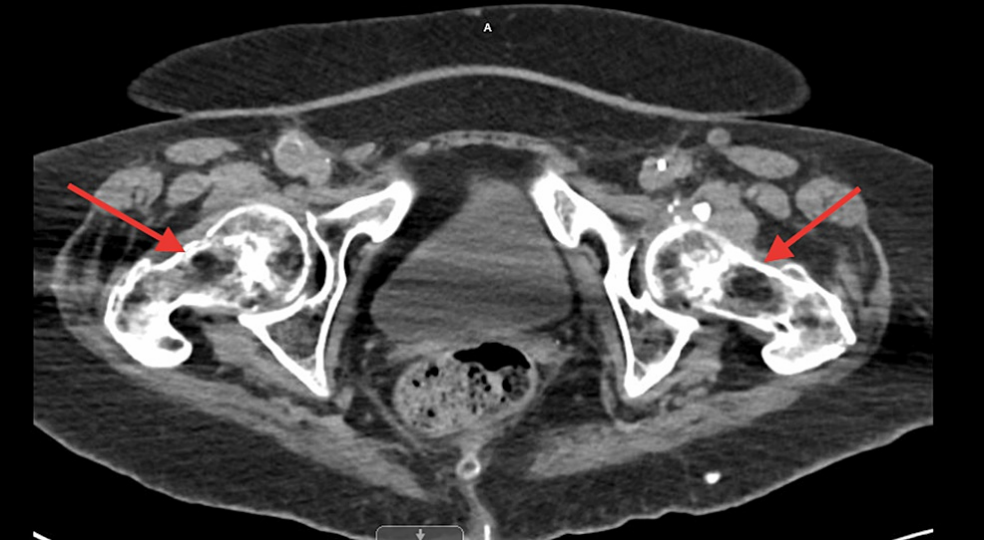
A CT angiogram of the abdomen showing heterogeneous masses in both proximal femurs, consistent with bony metastases

A 72-year-old African American female, a nursing home resident with multiple comorbidities, presented to the emergency department with altered mental status. The patient was obtunded, intubated, and placed on mechanical ventilation for airway protection. Initial laboratory results were significant for lactic acid 2.4 mmol/L, WBC 12.2 x103/uL, hemoglobin 15.1 g/dL, hematocrit 50.1%, and MCV 104.6 fL. Influenza A/B, respiratory syncytial virus, and coronavirus disease 2019 (COVID-19) polymerase chain reactions (PCRs) were negative. Blood cultures, urinalysis, and sputum culture with gram stains were unrevealing for any infectious activity. Chest X-ray revealed small bilateral effusions (Figure 1). At the time of admission, a computed tomography (CT) scan of the head without contrast showed no signs of an acute intracranial abnormality. A CT angiogram of the abdomen and thorax revealed a stable type B aortic dissection (Figures 2-3), and there were heterogeneous masses in both proximal femurs, consistent with bony metastases (Figures 4-5).

The patient required an extended intensive care unit stay due to multiple unsuccessful ventilatory weaning attempts. Otolaryngology was consulted for tracheostomy. Intraoperatively, they discovered a sizable left thyroid mass pushing the trachea to the right side. As a result, a left hemithyroidectomy was done.

**Figure 6:**
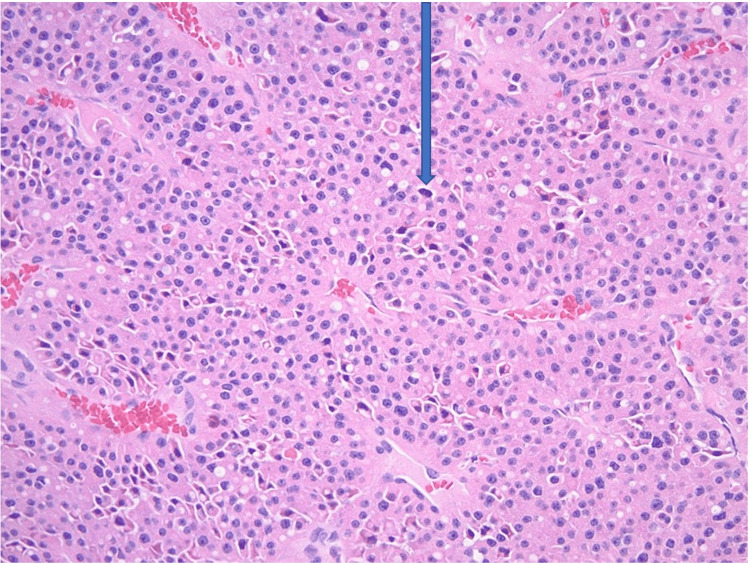
Oncocytic carcinoma of the thyroid, 100x The tumor is composed entirely of large tumor cells with abundant eosinophilic cytoplasm, arranged in solid architecture separated by delicate vasculatures.

**Figure 7:**
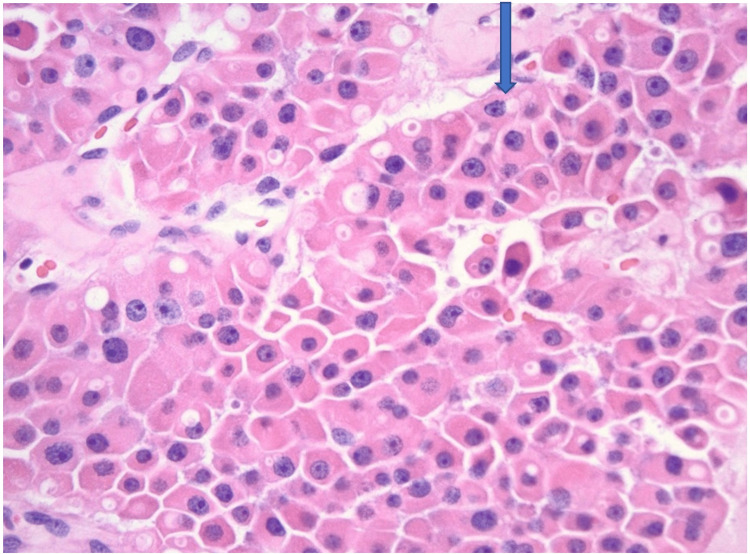
Oncocytic carcinoma of the thyroid, 400x The tumor cells are large, polygonal in shape with abundant eosinophilic granular cytoplasm, large round nuclei, vascular chromatin, and prominent nucleoli.

**Figure 8:**
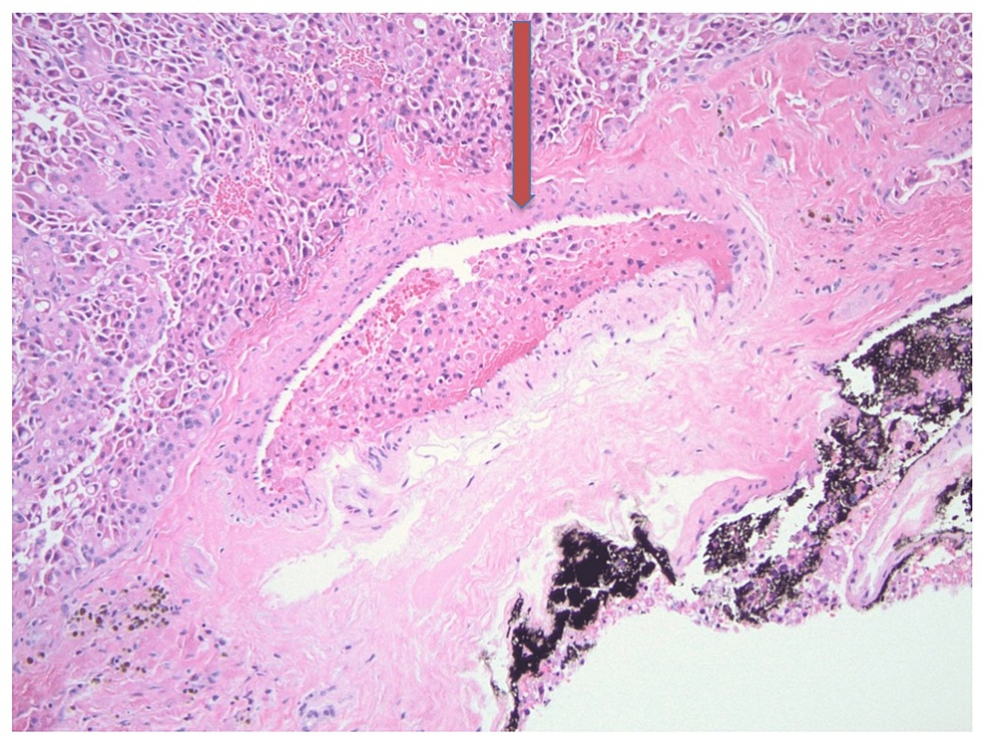
Oncocytic carcinoma of the thyroid, 100x The tumor cells are seen within a medium-sized vein.

Multiple nodules were found in thyroid tissue, with sizes ranging from 1.5 cm to 7 cm. Microscopically, the largest nodule was composed entirely of oncocytic cells - large polygonal cells with abundant eosinophilic granular cytoplasm, large round nuclei, vesicular chromatin, and prominent nucleoli. They were arranged in thick trabeculae and solid nests, separated by delicate vasculatures. Focally, tumor cells were seen within a medium-sized vein. See Figures 6-8. The above morphologic features were consistent with OCA. After a definitive diagnosis, a total thyroidectomy was performed. Thyroid profile revealed normal TSH (4.20 micro IU/dl), free T4 (0.93 ng/dl), free T3 (2.7 pg/ml) but elevated total T4 (13.8μg/ml), and decreased total T3 (70 ng/ml). Subsequently, a total thyroidectomy was done, and hematology-oncology was consulted. However, the patient declined further cancer care and requested changing care goals limited to comfort measures. As a result, she was discharged to a long-term care facility with thyroid hormone supplementation.

## Discussion

Histopathologically, they are benign if there is no evidence of vascular or capsular invasion and malignant if the invasion is present [2]. Oncocytic metaplasia occurs in many body organs due to various cellular stress responses. Oncocytic metaplasia can be seen in benign adenomatous nodules, Hashimoto thyroiditis, PTC, medullary, and poorly differentiated thyroid carcinoma. Oncocytic follicular cell-derived thyroid carcinomas can include many different entities: Oncocytic PTC, oncocytic encapsulated follicular subtype of PTC, oncocytic poorly differentiated carcinoma, and oncocytic medullary thyroid carcinoma [6]. No immunostains can distinguish OCA from oncocytic adenoma.

The definitive diagnosis of OCAs can be made by post-excisional histopathological analysis of the entire capsule [8]. OCA is diagnosed when a tumor consists primarily of oncocytic cells (> 75%), which originate from the follicular epithelium of the thyroid gland, and capsular invasion or vascular invasion is present, which is seen in our patient [9].

OCA is classified as minimally invasive, encapsulated with vascular invasion, and widely invasive. The minimally invasive terminology is restricted to tumors showing only tumor capsule invasion, whereas the encapsulated tumors showing vascular invasion are further subdivided into tumors with limited (less than four vessels) or extensive (equal to or greater than four vessels) vascular invasion. The microscopic features of concern include tumor necrosis, numerous mitoses (≥ 3/mm^2^), abnormal mitoses, and foci of small cell (cytoplasmic loss) change. Tumors with these features are likely to be radioiodine-resistant. Mutations typical of thyroid cancer genes, including RAS, TSHR, EIF1AX, TP53, PTEN, BRAF, PAX8-PPAR - Gamma, and MEN1, are uncommon in OCA [10].

Other methods of diagnosing thyroid neoplasms include cytologically by fine-needle aspiration (FNA), radiologically by ultrasound (USG), and occasionally by computed tomography (CT). However, these methods do not always provide a definitive diagnosis. For example, in a study by Dean DS and Hossein G, FNA biopsies revealed 65% benign, 5% malignant or suspicious for malignancy, 10% non-diagnostic, and 20% indeterminate [11].

The Bethesda system is widely used to report thyroid nodule cytology results with more uniform terminology. It is a six-tiered classification system with the following components: non-diagnostic or unsatisfactory, benign, atypia of undetermined significance/follicular lesion of unknown significance (AUS/FLUS), follicular neoplasm, suspicious of malignancy, and malignant. If the cytology reveals either follicular neoplasm or is suspicious for malignancy or positive for malignancy, surgery is recommended following preoperative evaluation [5].

In a case report by Rajmonda et al., a 51-year-old female patient presented with a visible mass on the neck while our patient, who is also a female, presented with acute encephalopathy at 72 years [12]. In a study by Viar JR et al., a 55-year-old female patient presented with severe back pain: progressive lower leg motor, and sensory weakness. She was found to have a T7 vertebral mass. Biopsy revealed metastatic follicular variant or PTC. A neck ultrasound disclosed well-defined calcified nodules in both thyroid lobes, she underwent total thyroidectomy, and a biopsy revealed minimally invasive OCA [13]. In our patient, the presentation was acute mentation changes, which are unique, and no symptoms in the lower extremities; on further history, she was known to be bedridden due to extreme debility and weakness. CT angiogram of the abdomen was suggestive of bilateral bony metastasis to the femur. Our patient has an unusual presentation at the age of 70s presented with no concerns of visible mass. Incidentally, during tracheostomy, the surgeon was suspicious of a large mass pushing the trachea. Patients' wishes were honored, and comfort care was offered. All further aggressive management was deferred.

Staging of OCAs

A staging system is an approved and conventional method for describing a tumor's size and spread. The American Joint Committee on Cancer (AJCC) described the TNM (tumor, nodes, and metastasis) system as the most widely used system for classifying thyroid cancer stages. The TNM approach is based on three essential data points: T denotes the primary tumor size, proportion, and growth into nearby tissues. N describes the extent of the tumor spread to nearby lymph nodes. M indicates whether cancer has metastasized to other organs of the body. Lungs, liver, and bones are the most prevalent locations of thyroid cancer metastasis [8].

Prognosis

OCAs tend to be aggressive, with a high risk of metastasis and a low survival rate. Its presentation ranges from encapsulated minimally invasive tumors to highly aggressive tumors (which have extensive vascular and extrathyroidal invasion). Patients with encapsulated minimally invasive OCAs, with microscopic capsular penetration and no vascular invasion, typically have a favorable prognosis [3,14,15]. Prognosis largely depends on the extent of vascular invasion, with the more vessels invaded, the more guarded the prognosis (mortality >90% at 10 years).

Other prognostic markers include age, gender, cancer stage, and degree of spread. The unfavorable prognostic factors include advancing age, male sex, larger tumor size at diagnosis, extra-thyroid expansion, and advanced stage at diagnosis [16,17]. In a retrospective study of 263 patients by Lukovic J et al., both oncocytic papillary thyroid carcinoma and oncocytic poorly differentiated thyroid carcinoma had higher 5- and 10-year incidence of locoregional failure and distant metastasis with an even higher incidence in the later [18]. Another study by Gross M showed the involvement of cervical lymph nodes in 43.4% of the patients [19].

Treatment

The management of thyroid cancers remains controversial. Areas of uncertainty include the indications for radioiodine ablation, timing, type of follow-up investigations, indications and techniques for external beam radiotherapy, and duration of hormonal suppression [20]. The treatment of OCAs can be classified into surgical and non-surgical approaches. Surgical intervention is the mainstay treatment for OCAs. Multiple studies and case series have demonstrated that thyroid lobectomy and total thyroidectomy had comparable results and efficacy in matched individuals with noninvasive tumors. In contrast, total thyroidectomy is the treatment of choice for invasive tumors [21].

Surgery

Surgical removal of the primary tumor is the main objective in the initial phase of treatment for OCAs. Thyroidectomy facilitates postoperative radioactive iodine treatment, and adequate treatment reduces the likelihood of recurrence. Surgical dissection of cervical lymph nodes is dependent on the patterns of disease spread and cancer stage [20,22]. Larger tumors (>T2) require total thyroidectomy and lymphadenectomy if lymph nodes are involved. There are three fundamental types of thyroidectomy: thyroid lobectomy entails the whole excision of a lobe as well as its isthmus. Subtotal/near-total thyroidectomy is thyroidectomy omitting the institution-recommended selected or comprehensive dissection, leaving remnant tissues, and total thyroidectomy with no remnant tissue.

TSH Suppressants

They are typically administered after surgery. Following total thyroidectomy, thyroxine is necessary to restore thyroid hormones and suppress TSH levels. Larger doses of thyroxine inhibit stimulation of the pituitary gland, hence preventing stimulation of the remaining thyroid tissue [20].

Iodine 131 or Radioiodine Therapy (RAI)

The fundamental notion underlying the use of radioactive iodine is that it quantifies the amount of residual cancerous thyroid tissue following ablation therapy. Normal thyroid tissue absorbs more radioactive iodine than cancerous thyroid tissue, aiding differentiation.

RAI iodine 131 helps in the earlier detection and treatment of metastatic disease; aids in interpreting serum thyroglobulin (Tg) measurements during serial follow-up. It aids in obliterating the thyroid remains [20].

Radioactive iodine (RAI) is used in the adjuvant setting for patients with high-risk characteristics such as tumor size >2 cm, positive margins, cervical lymph node metastases, microvascular invasion, or postoperative thyroglobulin levels > 1 ng/mL. However, only around 10% of patients with OCAs are likely to undergo radioactive iodine therapy; hence, responses to RAI therapy are substantially lower in these individuals compared to other forms of thyroid carcinomas [23].

Risks and toxicity of iodine 131: The adverse effects profile of radioactive iodine therapy consists of local effects: Neck pain, inflammatory reaction, altered taste, and sialadenitis; generalized effects: Dysfunction of the lacrimal gland, radiation cystitis, gastritis, bleeding diathesis, suppression of the bone marrow, infertility, and secondary malignancies.

Chemoradiation with External Beam Radiation Therapy (EBRT)

It is rarely utilized, but the most typical indications for EBRT are unresectable disease, gross local invasion with macroscopic or microscopic residuals/remnant cells, recurrent neck disease that is inoperable, palliation of inoperable metastases, and disease spread to the spine.

Radiation therapy may decrease RAI uptake in residual thyroid tissue; therefore, RAI administration should be considered prior to EBRT. Patients with de novo or residual cervical illness may be candidates for neck dissection if feasible. Additionally, intensity-modulated radiotherapy or external beam radiation therapy may be considered if surgical resection is not feasible or is contraindicated [20,24,25]. Chemotherapy and radiation are often considered as last resort for treating OCAs.

Chemotherapy and Biological Agents

Currently, there are insufficient data to support the use of adjuvant chemotherapy in treating differentiated thyroid tumors such as OCAs. Doxorubicin is the drug of choice in advanced thyroid cancer that has been investigated the most [26]. If the tumor is resistant to RAI, systemic treatment with tyrosine kinase inhibitors (TKI), such as lenvatinib or sorafenib, is recommended [27,28]. However, no conclusive evidence exists that it is associated with improved survival rates [29].

## Conclusions

OCAs are aggressive tumors with an exceedingly low incidence, a high risk of metastasis, and a poor prognosis. In our patient, OCA was detected as an incidental finding with the presence of bony metastases, and its clinical suspicion during tracheostomy itself is a unique presentation. Due to our patient's preference, cancer care was declined. Clinicians must keep a high index of suspicion of the possibility of OCA when appropriate. Unfortunately, OCAs have low overall survival benefits.
